# The impact of dose of the angiotensin-receptor blocker valsartan on the post-myocardial infarction ventricular remodeling: study protocol for a randomized controlled trial

**DOI:** 10.1186/1745-6215-12-247

**Published:** 2011-11-22

**Authors:** Young-Rak Cho, Young-Dae Kim, Tae-Ho Park, Kyungil Park, Jong-Sung Park, Heekyung Baek, Sun-Young Choi, Kee-Sik Kim, Taek-Jong Hong, Tae-Hyun Yang, Jin-Yong Hwang, Jong-Seon Park, Seung-Ho Hur, Sang-Gon Lee

**Affiliations:** 1 Department of Internal Medicine, Dong-A University College of Medicine, Busan, Korea; 2 Department of Cardiovascular Center, Dong-A University Hospital, Busan, Korea; 3 Department of Internal Medicine, Daegu Catholic University College of Medicine, Daegu, Korea; 4 Department of Internal Medicine, Pusan National University College of Medicine, Busan, Korea; 5 Department of Internal Medicine, Inje University College of Medicine, Pusan Paik Hospital, Busan, Korea; 6 Department of Internal Medicine, Gyeongsang National University College of Medicine, Jinju, Korea; 7 Department of Internal Medicine, Yeungnam University College of Medicine, Daegu, Korea; 8 Department of Internal Medicine, Keimyung University College of Medicine, Deagu, Korea; 9 Department of Internal Medicine, Ulsan University College of Medicine, Ulsan, Korea

**Keywords:** Valsartan, Myocardial Infarction

## Abstract

**Background:**

Angiotensin-converting enzyme inhibitors and the angiotensin-receptor blocker valsartan ameliorate ventricular remodeling after myocardial infarction (MI). Based on previous clinical trials, a maximum clinical dose is recommended in practical guidelines. Yet, has not been clearly demonstrated whether the recommended dose is more efficacious compared to the lower dose that is commonly used in clinical practice.

**Method/Design:**

Valsartan in post-MI remodeling (VALID) is a randomized, open-label, single-blinded multicenter study designed to compare the efficacy of different clinical dose of valsartan on the post-MI ventricular remodeling. This study also aims to assess neurohormone change and clinical parameters of patients during the post-infarct period. A total of 1116 patients with left ventricular dysfunction following the first episode of acute ST-elevation MI are to be enrolled and randomized to a maximal tolerable dose (up to 320 mg/day) or usual dose (80 mg/day) of valsartan for 12 months in 2:1 ratio. Echocardiographic analysis for quantifying post-MI ventricular remodeling is to be conducted in central core laboratory. Clinical assessment and laboratory test are performed at fixed times.

**Discussion:**

VALID is a multicenter collaborative study to evaluate the impact of dose of valsartan on the post-MI ventricular remodeling. The results of the study provide information about optimal dosing of the drug in the management of patients after MI. The results will be available by 2012.

**Trial registration:**

NCT01340326

## Background

Progressive enlargement of the heart chamber and deterioration of contractile function after myocardial infarction (MI), termed post-MI ventricular remodeling, is associated with development of heart failure and poor prognosis [1-3]. The magnitude of post-MI remodeling is influenced by several determinants, most notably infarct size [4], but also by ventricular wall stress [5], patency of infarct-related artery [6], and a number of neurohormonal factors [7]. Thus, the consequence of post-MI remodeling varies among patients with acute MI even in the era of reperfusion therapy [8]. Modification of neurohormonal acitivities, particularly the rennin-angiotensin-aldosterone system (RAAS), can significantly influence the process of ventricular remodeling after acute MI. Suppression of angiotensin activity either by inhibition of angiotensin-converting enzyme (ACE) [9-11] or by blockade of angiotensin II receptor [12] attenuates ventricular dilatation and improves clinical outcomes. Based on the results from major pivotal clinical trials, it is usually recommended in practical guidelines that the maximal clinical dose of ACE inhibitors used in those trials be given to patients after acute MI [13,14]. A seminal finding that the neurohormone level is linearly related with mortality [15] in patients with heart failure also suggests the potential benefit of higher doses.

However, the optimal level of RAAS antagonism in the treatment of heart failure or post-MI remodeling is still a matter of debate. Although administration of higher dose of angiotensin-converting enzyme (ACE) inhibitor was more beneficial than lower dose in animal model of post-MI remodeling [16], results of clinical studies were not confirmatory. In the VALIANT study [17], addition of ARB valsartan to ACE inhibitor resulted in similar degree of post-MI left ventricular (LV) remodeling compared to either drug alone, although in the Val-HeFT study [18], LV remodeling in heart failure was more favorable in the combination therapy group. In several clinical studies that directly compared different doses of ACE inhibitors in patients with chronic heart failure, the results of clinical outcomes as well as neurohormonal responses were inconsistent, and sometimes irrelevant because of the impractical dosing of drugs chosen for the comparison. In the ATLAS study [19], patients receiving high-dose lisinopril (32.5-35.0 mg/day) had a nonsignificant (8%) lower risk of death and a significant (12%) lower rate of death and hospitalization compared with patients receiving low-dose lisinopril (2.5-5.0 mg/day). The CHIPS trial [20] compared low-dose (50 mg/day) with high-dose (100 mg/day) captopril therapy and demonstrated a nonsignificant trend toward less worsening heart failure and hospitalization in the high-dose group. In the NETWORK trial [21], there was no difference in the primary endpoint of combined death, heart failure-related hospitalization, and worsening of heart failure among the three groups of low dose (5 mg/day), medium dose (10 mg/day), and high dose (20 mg/day) enalapril therapy. Furthermore, the lower dose used in NETWORK (enalapril 5 mg/day) [21] was too small, and the higher dose used in ATLAS (lisinopril 35 mg/day) [19] was excessive for practical use. The recent publication of the Heart failure Endpoint evaluation of Angiotensin Antagonist Losartan trial (HEAAL) presented notable evidence of the superiority of 150 mg/day of losartan versus 50 mg per day on the primary outcomes of death or hospitalization in patients with systolic heart failure [22]. However, the study subjects were limited to patients who were intolerant to ACE inhibitors [22], precluding extrapolation of the result to general patients. Furthermore, the optimal dosing of ARB agents has not been explored in the population of post-MI. Thus, the question of whether submaximal dose of ARB, which are lower than those in major pivotal trials but typically used in clinical practice, can offer similar benefit in post-MI ventricular remodeling remains to be solved. This is more so in the Asian population, wherein moderate dose ARB has been shown to provide sufficient protection from cardiovascular risk [23]. Therefore, the primary objective of the VALsartan in post-mI remoDeling (VALID) study is to address this issue in Korean patients who suffered their first acute ST-elevation MI by comparing the impact of different doses of valsartan, an ARB demonstrated as effective as ACE inhibitor in post-MI patients [12], on echocardiographic variables of left ventricular remodeling during the follow-up period of 1 year. The comparison dose of valsartan will be 80 mg per day, as commonly prescribed after MI in Korea, versus 320 mg per day, a targeted dose in major clinical trials [12,24].

## Trial objectives

The primary hypothesis to be tested is whether high dose valsartan significantly reduces ventricular remodeling as measured by echocardiography in post-MI patients during a 12-month follow-up period, compared with the usual dose of control group.

## Methods and Design

### Study design

**Figure 1:**
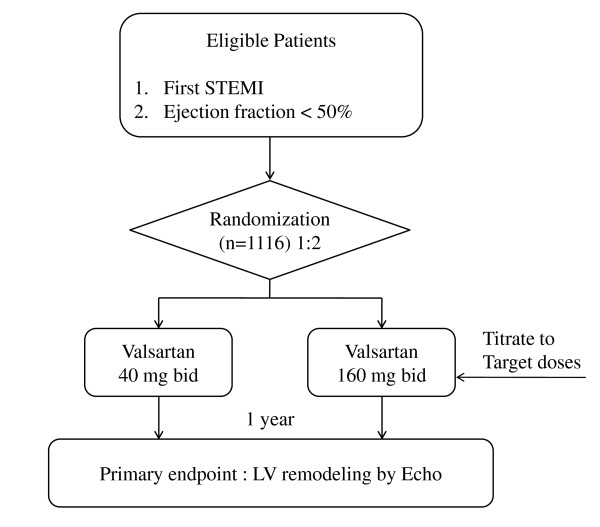
**Overall study algorithm of the VALID study**. This figure illustrates the study algorithm. A total of 1116 patients will be randomly allocated into the usual dose group (n = 372) and high dose group (n = 744) and followed-up for 12 months after discharge.

VALID is a prospective, multicenter, randomized, open-label, active controlled study with two parallel study groups. VALID is being conducted in 18 tertiary hospitals throughout South Korea. Participants are randomly allocated into the usual dose group and high dose group and followed-up for 12 months after discharge. Overall study algorithm is depicted in Figure 1. Baseline echocardiographic examination and neurohormonal assay are performed before discharge. Study approval was given by the institutional review board at each participating center, and consecutive, eligible patients are provided written informed consent.

### Randomization

Randomization will take place following initial echocardiographic estimation of left ventricular ejection fraction, which is conducted after stabilization by reperfusion therapy or conservative treatment. Eligible patients are randomly assigned in a 1:2 ratio to receive the usual dose (valsartan 80 mg/day) or a high dose (valsartan up to 320 mg/day). Random allocation with stratified technique is generated automatically by a centralized web based tool (http://www.cnrres.co.kr/valid) so it cannot be influenced by researchers.

### Approval

This study follows the Helsinki Declaration's principles, meaning that all patients sign a written informed consent stating that participation is voluntary and that participation can

be withdrawn at any time, without any negative consequences concerning their current or future medical treatment. This study protocol was approved by the institutional review board of Dong-A University hospital and each participating center.

### Patient population

Men and women aged 18 years or older who suffer their first acute ST-elevation myocardial infarction with the sign of LV dysfunction are eligible for this study (Table 1). In the present study, LV dysfunction is defined as an ejection fraction < 50% using a modified Simpson's rule [25]. Enrollment criteria are intended to include patients in acute stage of infarction, within 10 days of symptom onset, who are typically treated under modern therapeutic strategy. Patients are recruited regardless of whether they received reperfusion therapy, either thrombolysis or primary percutaneous coronary intervention, or not. Pharmacological therapy other than study drug, including beta-adrenergic blockers, is allowed according to the discretion of attending physician. Patients who have contraindication to the study drug or major concomitant disease are excluded (Table 1).

**Table 1:** Eligibility criteria

Inclusion criteria	Exclusion criteria
Subjects > 18 years of age	Contraindication for use of ARB

Either gender	Urgent need for revascularization procedure

First episode of acute MI	Severe heart failure (NYHA IV or need for inotropic support)

Typical pain lasting ≥ 20 minutes	Persistent (>1 hour) severe hypotension (systolic blood pressure < 90 mmHg)

ST elevation of more than 1 mm in at least 2 separate leads on the ECG	Refractory or potentially lethal arrhythmias

An echocardiographic LV EF < 50%	Hemodynamically significant right ventricular infarction

Optimal recording of echocardiographic imaging of apical chamber views	Congenital heart disease

Patients who provide written informed consent	Primary valvular disease, severer than mild degree

	Idiopathic hypertrophic cardiomyopathy

	Concomitant inflammatory cardiomyopathy

	Significant renal dysfunction (serum creatinine 2.5 mg/dl)

	Significant hepatic dysfunction (serum transaminase more than 3 times normal)

	Anemia (hemoglobin < 10 mg/mL or 6 mmol/L)

	Psychiatric disorders, alcohol or drug abuse

	Life expectancy is less than 1 year

	Participation in any other pharmacological study within 2 months

	Refusal or inability to provide informal consent

### Intervention and comparator descriptions

**Figure 2:**
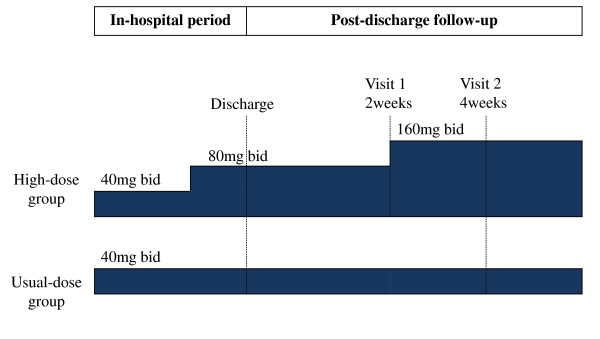
**Titration scheme of study drug**. In the usual dose group, valsartan 40 mg twice a day is administrated throughout the study period. For those in the high dose group, dose is up-titrated to 80 mg twice a day before hospital discharge and finally to 160 mg twice a day after 2 weeks during outpatient visits.

Eligible patients are randomly assigned in a 1:2 fashion to either the usual dose group (valsartan 80 mg/day) or the high dose group (valsartan up to 320 mg/day). In the usual dose group, valsartan 40 mg twice a day is administrated throughout the study period. For those in the high dose group, dose is up-titrated to 80 mg twice a day before hospital discharge and finally to 160 mg twice a day after 2 weeks during outpatient visits. The process of the trial conduct is illustrated in Figure 2. If up-titration is not clinically feasible, either because of hypotension or deepening azotemia, the previous dose is administered subsequently as maximal tolerable dose.

### Outcomes measurement

The primary outcome of the study is the changes of the echocardiographic indices of LV remodeling, which is the LV volume index at end-systolic and end-diastolic time as measured by modified Simpson's rule [25], during the study period. Echocardiographic records from participating institute are sent to the core echocardiography laboratory for the analysis. Secondary outcomes include occurrence of predefined clinical events (all-cause death, cardiovascular death, hospitalization, revascularization), changes of plasma level of neurohormone (B-type natriuretic peptide, norepinephrine, aldosterone), and echocardiographic indices other than ventricular volume (ejection fraction, wall motion score index, mitral inflow, tissue Doppler index) at 12 months (Table 2). Neurohormone measurement will be conducted in a core laboratory.

**Table 2:** Study Objectives

Primary Objective	Secondary objectives
Change in the LV index measured by echocardiography from baseline to follow-up	Change in functional capacity (NYHA class)

	All cause mortality

	Cardiovascular death

	Hospitalization

	Revascularization procedures (emergency and elective)

	Change in B-type natriuretic peptide (BNP) level

	Change in plasma norepinephrine level

	Change in serum aldosterone level

	Change in ejection fraction

	Change in MI index (wall motion score index)

	Change in sphericity index

	Change in mitral inflow index (mitral E/A ratio, mitral deceleration time)

	Change in tissue Doppler index (mitral Ea)

### Follow-up protocol

During the study period, study visits are scheduled at week -2 (high-dose group only), and 1, 3, 6, 9, 12 months. At each visit, patients undergo a complete physical examination, medical history-taking, and assessment of drug compliance. Investigators evaluate all clinical and laboratory adverse events at each visit. To monitor safety, serum creatinine and urea nitrogen concentrations are determined at every study visit. New York Heart Association (NYHA) functional class [26] and predefined clinical events are recorded at each clinical visit. Echocardiographic examination and neurohormonal assay are performed at 3 and 12 months after discharge. After a routine review with 50% of the patients enrolled, early cessation of the trial will be decided by the institutional review board and study sponsor when the trial appears to be futility or causing unexpected harm to participants.

### Adverse effects

Analysis of safety related data is performed with respect to frequency of serious adverse events, stratified by causality and intensity of morbidity in both treatment groups.

Patients are interviewed at each visit about the occurrence of any adverse events, including the time of onset, duration, and severity; all information is recorded on a case report from. The causal relation to the study drug and the intensity of adverse events are evaluated by the investigators. Serious adverse events (SAE) have to be reported to the institutional review board and study sponsor by the principal investigator within 24 hours after the SAE becomes known.

### Withdrawals

Patients are free to withdraw trial participation at their own request at any time and without giving reasons for their decision. Moreover, the primary investigator can withdraw study patients, if continuation of the trial would be detrimental to the patient's

well being. Withdrawals will be documented in the case report form and in the patient's medical records and all ongoing severe adverse event have to be followed up.

### Sample size

The sample size calculation is based on the primary outcome and the primary analysis for the intention-to-treat population. In the VALIANT Echo study, [17] a sample size of 600 patients was determined necessary to detect, with a 90% power, a 7.6 mL difference in end-diastolic volume between the treatment groups. Because the present study enrolls more patients with mild degree (EF < 50%) LV dysfunction, we anticipate that the difference of ventricular volume between treatment groups will be smaller than the previous study. Thus, detection of 3.8 mL difference, with a two-sided level of significance α = 5% and a power of 1-β = 90%, in end-diastolic volume between treatment groups would require a sample size of 279 patients in a usual dose of valsartan group and 558 patients in a high dose valsartan group. Assuming 25% of patients are lost to follow-up or with missing data, as reported in the GISSI-3 study [27], the required sample size of this study will be a total of 1,116 patients. The study is likely to be underpowered in terms of assessing secondary outcomes if this size is employed. Variability data for the outcome measures could be used to inform design a subsequent larger-scale randomized controlled trial, if post hoc analysis reveals this study to be underpowered.

### Statistical analyses

A consultant group of statisticians has been appointed to conduct the statistical work for this study, including data completion, interim analysis, application of statistical technique and final assessment. The principal analysis will be an intention-to-treatment basis. For baseline characteristics, continuous variables are assessed using the Student *t *test and discrete variables are compared using the chi-square test. The two-sided null-hypothesis for the primary outcome measure states that usual and high dose valsartan lead to the same expected change of ventricular remodeling during the 12 months after MI. This null-hypothesis will be tested by application of an analysis of covariance that adjusts for age, gender, and cardiovascular risk factors. Primary outcome will be compared using 2-sample *t *tests. Event-free cumulative survival rates are plotted using the Kaplan-Meier method and comparisons are made between patients with and those without clinical events using the log-rank test. A Cox proportional hazards model with the use of forward selection based on the likelihood ratio test will be implemented for multivariate analysis to determine which prognostic factors identified in the univariate analysis were significantly related to 12-month clinical events. Assessments for the change of neurohormone and other echocardiographic indices will be compared between treatment groups at 12-month follow-up using 2-sample *t *tests.

We will compare proportions of missing data using chi-square tests and agreement between data collection methods using Lin's Concordance Correlation Coefficient. Additionally, sensitivity analyses will be conducted using different patient populations (per protocol population excluding patients with relevant protocol violations), different imputation techniques for missing values, and different statistical methods for taking into account covariates. A value of p < 0.05 will be considered statistically significant.

## Discussion

Despite the accepted roles of ACE inhibitors and the ARB agent valsartan in the treatment of post-MI LV dysfunction [9-12], the appropriate dose remains unclear. Although clinical guidelines recommend to prescribe the doses that have been shown to reduce the risk of cardiovascular events in clinical trials [13,14], it is common practice for the patients to be maintained on doses appropriate for initiation of therapy rather than doses up-titrated to target doses used in the clinical trials. Concerns about patient's intolerance to higher doses might be an attributable factor for such practice. Although the recent HEAAL study demonstrated a superiority of higher dose over low dose losartan in ACE intolerant patients with heart failure, the evidence is still lacking for the setting of post-MI LV dysfunction and for general population. The VALID study has been designed to address the issue of optimal dosing of ARB valsartan in the post-MI patients by comparing the major echocardiographic outcome of post-MI LV remodeling, which is the LV volume index. Because the differences of clinical outcomes between high- versus low-dose therapy is expected to be considerably smaller than those of placebo-controlled landmark trials, quantitative measurement of post-MI ventricular remodeling can be a reasonable surrogate endpoint.

## Conclusions

The enrollment criteria for the VALID is different from those of other major post-MI ACE inhibitor trials in that patients with milder systolic LV dysfunction (EF <50%) is eligible for VALID while more severe form of LV dysfunction (EF <35% or clinical sign of heart failure) was required for enrollment in previous major clinical trials [9-12]. Two factors have been considered when deciding the cut-off value for LV dysfunction. First, the majority of patients referred to the participating hospital receive reperfusion therapy in Korea [28], leading to substantial increment of the number of the patients with more preserved LV contractile function. Second, as the proportion of elderly population among the patients with acute MI is increasing, the pattern of post-MI remodeling may exhibit a different picture. Elderly patients seem to undergo LV remodeling more commonly than younger patients, even after smaller size acute MI. In the PREAMI study [29], ACE inhibitor (8 mg/day of perindopril) could reduce progressive LV remodeling in elderly patients who had a relatively preserved LV function with EF ≥40%. Thus, the increasing proportion of elderly population in acute MI cases in Korea [30] can make it feasible to examine the effect of ARB agent on post-MI remodeling in the presence of moderate LV dysfunction for the VALID study.

## List of abbreviations

ACE: Angiotensin-converting enzyme; MI: Myocardial infarction; LV: Left ventricle; EF: Ejection fraction; RAAS: Renin-angiotensin-aldosterone system; VALID: Valsartan in post-myocardial infarction remodeling; βAR: Beta-adrenergic blocker; ARB: Angiotensin II receptor blocker.

## Competing interests

The authors declare that they have no competing interests.

## Authors' contributions

YDK is the Principle Investigator for the study, contributed to the study design and to drafting and revising the manuscript. YRC made significant contribution to concept of the study, drafting and reviewing manuscript. All authors read and approved the final manuscript.
